# Exploring the Impact of Perceived Parental Oversight on Problematic Smartphone Use Among Adolescents in the Digital Age: Database Analysis

**DOI:** 10.2196/75837

**Published:** 2025-12-04

**Authors:** Mary Ho, Peter Johannes Schulz, Angela Chang

**Affiliations:** 1 Institute of Communication and Public Policy, Faculty of Communication, Culture and Society, Università della Svizzera Italiana, Lugano, Switzerland; 2 Department of Public Health and Medical Administration, Faculty of Health Sciences, University of Macau, Taipa, Macao, China; 3 Wee Kim Wee School of Communication and Information (WKWSCI), Nanyang Technological University, Singapore, Singapore; 4 Department of Communication, Faculty of Social Sciences, University of Macau, E21-2056, Avenida da Universidade, Taipa, 100, Macao, China, 853 88228991

**Keywords:** problematic smartphone use, parental monitoring strategies, adolescent digital behavior, developmental differences, socioeconomic factors, mobile phone

## Abstract

**Background:**

The proliferation of smartphones raises worries over their impact on adolescent development, especially problematic smartphone use. This research investigates the intricacies of problematic smartphone usage in adolescents, particularly in light of significant increases in screen time, from a developmental psychology perspective.

**Objective:**

This study aimed to examine the association between adolescent-perceived parental monitoring strategies and problematic smartphone use in Taiwan, while also exploring variations across age, gender, and socioeconomic status.

**Methods:**

A nationwide dataset from Taiwan’s annual survey (n=1673; aged 10‐18 y) was analyzed using descriptive analysis and moderated multiple serial mediation regression. In total, 3 parental mediation styles—restrictive monitoring, evaluative mediation, and unfocused monitoring—were examined for their effects on smartphone usage.

**Results:**

Rigorous surveillance is more beneficial for younger adolescents (aged 10‐12 y), significantly reducing smartphone addiction. Conversely, as teenagers mature, the efficacy of restrictive approaches wanes. Adolescents aged 16-18 years benefit more from parental mediation strategies that foster autonomy and encourage appropriate digital conduct. Restrictive monitoring significantly diminishes addiction by constraining internet access; yet, the diverse outcomes of assessment methods highlight the importance of qualitative engagement. Conversely, unfocused surveillance is ineffective, necessitating the use of targeted parental strategies.

**Conclusions:**

This study highlights the significance of developmentally suitable parental strategies to mitigate digital addiction and enhance teenage self-regulation. We urge policymakers to implement age-specific, evidence-based methods to improve digital literacy and overall well-being in youth. Future research should investigate the enduring psychological and behavioral impacts of parental mediation and analyze cross-cultural differences in digital parenting methodologies.

## Introduction

### Background

In the digital era, smartphones have become indispensable tools for communication, education, and entertainment. Among adolescents, smartphone penetration is rapidly increasing, with more than 80% of individuals aged 12-17 years owning such devices [1]. While digital access fosters connectivity and learning, it also presents growing concerns about problematic smartphone use (PSU), characterized by compulsive checking, excessive screen time, and withdrawal symptoms upon separation [2,3]. PSU has been associated with disrupted sleep, impaired academic performance, and hindered emotional development, establishing it as a pressing public health issue.

Previous studies suggest rising smartphone addiction among adolescents, with prevalence rates ranging from 10% in the United Kingdom to more than 30% in South Korea [4,5]. Moreover, the COVID-19 pandemic significantly reshaped digital media usage. Remote learning, physical distancing, and prolonged home confinement have intensified adolescents’ reliance on digital devices for social interaction and emotional coping [6]. This shift has sparked increased risks of excessive use and internet addiction, with adolescents turning to social media and gaming to alleviate pandemic-induced stress and loneliness [7,8]. These behavioral shifts are particularly concerning given adolescents’ developmental vulnerabilities, such as immature impulse control and heightened sensitivity to peer influence [9].

Parental mediation is a critical factor influencing adolescent digital engagement and well-being. Empirical evidence consistently demonstrates that parental involvement is associated with a reduction in adolescents’ risky online behaviors and problematic media use [10,11]. Specifically, studies have established a significant, although complex, association between various parental mediation strategies (eg, restrictive, active, and technical mediation) and attitudes, as well as reduced levels of adolescent problematic media habits [12-15]. Strategies such as screen-time limits, content monitoring, and open communication are commonly used, yet the effectiveness of these measures may be mediated by adolescents’ perceptions of control and autonomy [15]. Thus, the parent-adolescent dynamic and the child’s perception of mediation are crucial variables determining its success.

Theoretical models like parental mediation theory emphasize that youth interpretations of parental actions, whether perceived as autonomy-supportive or controlling, determine their behavioral responses [16,17]. Recent studies continue to apply these models to examine how evolving parental strategies shape adolescents’ online experiences [18]. Although pioneering work has begun to effectively incorporate adolescent perspectives on parental mediation, particularly concerning social media use [19,20], research on smartphone-specific problematic use (PSU) continues to rely more heavily on parents’ self-reported practices. Consequently, the adolescent’s subjective experience of mediation strategies applied to the unique challenges of smartphone use remains less explored. This limits the development of tailored, context-sensitive approaches to digital well-being.

### Goal of This Study

This study aims to examine the association between perceived parental oversight and PSU among adolescents, guided by parental mediation theory. By integrating quantitative assessments of PSU with adolescents’ subjective interpretations of parental strategies, the research seeks to illuminate how different mediation styles influence digital behavior. The findings will inform interventions that align more closely with youths’ psychological needs, especially in contexts shaped by increased digital dependency, such as during public health crises. In doing so, this study positions parental oversight as a dynamic and influential factor in designing effective interventions for adolescent digital well-being.

### Literature Review

Adolescent media use significantly influences psychological well-being, with excessive screen exposure linked to body dissatisfaction, anxiety, and distraction [21-23]. During the COVID-19 pandemic, digital reliance intensified, exacerbating emotional vulnerability and compulsive behaviors [6,7]. While mobile technology offers educational and social benefits, unregulated use can harm development, particularly in environments lacking effective parental mediation [24,25]. Cross-national studies reveal varied addiction rates, underscoring sociocultural and familial influences [5]. Despite the known risks, few studies integrate youth perceptions of parental oversight into intervention frameworks. To address this, this study draws on 2 complementary theoretical frameworks. First, family systems theory posits that individual behavior, including media use, is shaped within the dynamics of the family unit. Parent-child interactions, including monitoring strategies, are seen as bidirectional and mutually influential, making parental mediation a relational, rather than merely regulatory, process [26,27]. Also, the positive youth development (PYD) framework emphasizes the importance of nurturing environments and adult support in fostering adolescents’ internal assets, such as self-regulation and resilience [28]. From this view, autonomy-supportive parental involvement can help adolescents build healthy digital habits. The existing research attempts to bridge the research gap by synthesizing family systems theory with PYD, positioning parental supervision as a dynamic moderator of self-regulated smartphone engagement (SRSE) [16,17]. Their approach reframes oversight not as a static constraint but as a modifiable factor that shapes the efficacy of SRSE-based interventions.

### PSU and Adolescent Health Risks

The increasing ubiquity of smartphones has reshaped adolescent behavior and well-being. While moderate digital engagement can foster social connection and access to diverse information [29,30], excessive and unregulated use contributes to a range of psychosocial challenges. PSU refers to a behavioral pattern characterized by compulsive use, impaired control, and continued use despite experiencing negative consequences in daily functioning [24,25,31]. Common manifestations include preoccupation with the device, withdrawal symptoms (eg, anxiety when separated from it), tolerance (needing increased use for satisfaction), and a significant disruption to academic, social, or sleep routines [32,33].

Adolescents are a particularly vulnerable population for PSU. Empirical studies have consistently linked PSU to adverse outcomes, including diminished mental well-being, reduced self-esteem, sleep disturbances, academic difficulties, and increased feelings of loneliness [23,34-37]. The COVID-19 pandemic intensified digital reliance, especially among youth, exacerbating digital overexposure and mental health strains [6,7]. In Taiwan, smartphone usage surpasses 94.9% [38], raising significant concerns about youth digital addiction. The prevalence of smartphone addiction in adolescents much surpasses that in adults, with estimates suggesting that 1 in 3 teenagers is at risk [38].

Previous studies explore the implications of smartphone use among different age groups, highlighting the associated health risks of PSU among adolescents. Park and Park [39] introduce a conceptual model of smartphone addiction specifically targeting early childhood, discussing factors that contribute to the development of compulsive smartphone behaviors and their potential impacts on young children’s social and cognitive development. In contrast, 1 study [40] conducted a cross-sectional survey focusing on middle- and high-school students in China, revealing the prevalence of PSU within this demographic and identifying significant correlations with mental health issues, academic performance, and social interactions. Together, these studies underscore the need for targeted interventions to mitigate the risks associated with excessive smartphone use and promote healthier digital habits among adolescents.

Sex-based differences also shape PSU patterns. While males more frequently engage in compulsive gaming and exposure to violent content [41], females report higher levels of internalizing symptoms, such as anxiety and depression, due to screen overuse [36,37]. These variations suggest the importance of tailored strategies in addressing PSU across demographic groups.

In addition, adolescents’ perceptions of parental oversight, whether controlling or autonomy-supportive, can significantly moderate the outcomes of interventions targeting smartphone habits [16,17]. As such, understanding PSU requires a multifaceted perspective that incorporates developmental, behavioral, and social determinants. Effective responses should consider not only individual behaviors but also relational factors, particularly the evolving role of parents in managing adolescents’ digital environments.

### Predicting Digital Behaviors

Parental monitoring has emerged as a key factor influencing adolescents’ digital engagement and their susceptibility to PSU [10,11]. Parental monitoring is traditionally defined as parental efforts to supervise and regulate children’s activities, including digital media use [42,43], and updated with reduced exposure to online risks such as violent content, gambling, and inappropriate interactions in the current digital era [44,45]. Scholars commonly categorize parental mediation into 3 distinct types. The first category is restricted mediation. This involves setting rules and limits governing screen time or access to specific apps and content [10,43]. Evaluative mediation, often referred to as educational or active mediation, involves parent-child discussions about media content aimed at fostering critical understanding and resilience [10,43,46]. Unfocused mediation describes co-use without explicit educational or restrictive intent, often characterized by shared but unsupervised media consumption [43]. One of the research gaps in the existing research usually relies on parents’ self-reported practices, overlooking the impact of adolescents’ subjective perceptions.

Recent studies highlight that the effectiveness of parental supervision hinges not only on behavioral enforcement but also on how adolescents interpret these efforts. Perceived overcontrol may elicit resistance, while autonomy-supportive practices foster self-regulation [16,17]. For instance, research in Taiwan found no significant association between parental monitoring and adolescent smartphone addiction, suggesting that the mere presence of supervision does not guarantee behavioral change [47]. Furthermore, some data show that heightened parental control may paradoxically increase digital risk-taking when perceived as intrusive [48,49].

Given the inconsistencies across contexts and measurement approaches, this study centers on adolescents’ perceived parental monitoring rather than parental self-report. By capturing children’s subjective experiences, this research seeks to clarify how parental regulation interacts with psychological factors to shape digital behavior, offering a more dynamic understanding of preventive strategies in adolescent technology use. Thus, the first hypothesis is raised, which states that adolescents’ perceptions of higher restrictive, evaluative, and unfocused parental supervision will be associated with lower levels of (1) daily internet usage and (2) PSU.

### Family Structures

#### Sex

Parents are anticipated to use distinct regulatory approaches with male as opposed to female children in accordance with the sex roles established within their society. Research suggests that parents use distinct control tactics based on the gender of their children, and the degree to which this occurs varies between fathers and mothers. For instance, research has shown that parents give less independence to their girls and protect them more than boys [50]. Moreover, research on adolescents’ media use of several kinds revealed that females were more likely than boys to have phone-using restrictions [51]. However, some research reported that parents tend to supervise their male children to a somewhat higher degree compared with their female children [52]. Some others have discovered that there is no discernible disparity in parental supervision based on the gender of the child [53].

Currently, there is a scarcity of evidence and inconclusive findings about the impact of parental mediation on cell phone addiction in children, specifically in relation to gender disparities. Based on the discussions made in the previous research, the objective of this study is to investigate the following research question (RQ) and propose one hypothesis to guide our analysis.

RQ1 was to examine how adolescents’ genders impact parental supervision and the prevalence of internet use and mobile phone addiction among teenagers.

Hypothesis 2 states that parental monitoring has a greater impact on girls’ intention to use the internet and their levels of mobile phone addiction compared with boys.

#### Age

Monitoring by parents elicits varied responses from children of different age groups. Not surprisingly, evidence regularly demonstrates that parents supervise the television and media consumption of their younger children to a greater extent than they do with their older children [54]. Furthermore, due to the concern that younger children are more prone to acquiring mobile phone addiction, research suggests that parents are more inclined to implement restrictive mediation and evaluative mediation with their younger children [39,53,55].

To go deeper into the studies of specific ages and the effect of parental monitoring differences, 1 study [56] done in Korea in 2015 proved that parents usually use restrictive mediation to monitor their kids in elementary school. A later study [57] in 2018 highlights that higher restrictive parental monitoring is correlated with a lower risk of mobile phone addiction among adolescents aged 11-13 years. Similar results were also found from a study by Chang and other researchers [47] who surveyed 2621 kids and 2468 parents from the main cities of Taiwan. They indicated that only the restrictive parental monitoring mediation could effectively decrease smartphone addiction risks among fifth-grade kids (aged 10-11 y). Accordingly, similar results are also found among middle-school and high-school kids (age range from 14 to 19 y old) in China, Japan, Thailand, and Portugal; their results suggested that parental neglect was significantly positively associated with adolescents’ mobile phone addiction, and perceived parental monitoring of smartphones was negatively related to adolescent mobile phone addiction [58-61].

The study investigates the threshold at which the efficacy of parental monitoring starts to decline or vanish to discover whether age is a significant mediator. Thus, RQ2 and another hypothesis (hypothesis 3) of the effectiveness of parental monitoring are raised:

RQ2 seeks an answer to whether parental monitoring has universal effectiveness across adolescents of varying ages, and at what age does the effectiveness of parental monitoring begin to diminish or disappear.

Hypothesis 3 states that younger adolescents experience higher levels of parental monitoring regarding their internet usage and mobile phone addiction compared with older adolescents.

#### Income and Parental Educational Level

Family socioeconomic status, particularly parental income and education, plays a significant role in shaping adolescents’ vulnerability to smartphone addiction. Previous research demonstrates that lower household income and parental education are associated with higher risks of problematic mobile phone use in adolescents [62,63]. A scoping review [64], synthesizing findings from 67 studies, supports this trend by identifying socioeconomic disadvantage as a consistent predictor of mobile phone addiction. Parents with limited education may lack awareness of the psychological risks associated with excessive smartphone use, increasing the likelihood of early and unrestricted access [65]. Financial constraints may also reduce parental capacity to offer alternative recreational or social resources, leading to greater screen dependence among youth [66]. Without access to structured extracurricular activities or supervised community spaces, adolescents from lower-income households may rely more heavily on smartphones for entertainment and socialization, thereby increasing their risk of problematic use.

The connection between socioeconomic status and parental mediation remains inconclusive. Previous studies suggest that adolescents from higher socioeconomic backgrounds may exhibit elevated levels of digital dependency, driven by feelings of isolation or pressure to remain socially connected via advanced devices [65,67]. This duality highlights the need for a nuanced understanding of how different economic and cultural contexts shape digital behaviors. While some research reports no significant association [68], others suggest that low-income families may favor restrictive or evaluative mediation strategies [69,70]. These inconsistencies underscore the complex interplay between structural constraints and parenting styles, emphasizing the need to account for family background when designing interventions for adolescent smartphone addiction. Thus, 2 RQs (RQ 3 and RQ 4) and 2 hypotheses are raised.

RQ3 aims to examine whether there is a relationship between household income and the practice of parental monitoring styles, and how this relationship affects adolescent mobile phone addiction. RQ4 aims to examine whether there is a relationship between parental education levels and the practice of parental monitoring styles, and how this relationship affects adolescent mobile phone addiction.

Hypothesis 4 states that higher household income is associated with more effective parental monitoring styles, which in turn reduces the vulnerability to mobile phone addiction among Taiwanese adolescents aged 10-18 years. Hypothesis 5 states that higher parental education levels are associated with more effective parental monitoring styles, which in turn reduces the vulnerability to mobile phone addiction among Taiwanese adolescents aged 10-18 years.

## Methods

### Ethical Considerations

Our study is a secondary data analysis based on the Taiwan Communication Survey (TCS), a nationally representative dataset administered by the Institute of Sociology, Academia Sinica (Taipei, Taiwan). The dataset was fully anonymized before public release and made available for academic research. The Academia Sinica Humanities and Social Science Research Ethics Committee reviewed and approved the project “Taiwan Communication Survey – Phase II, Year 4-5” under approval AS-IRB-HS-19027. This study used data from this approved project.

During the original data collection, written informed consent was obtained from all adolescent participants and their legal guardians before participation. To protect participants’ privacy, personally identifiable information was anonymized and restricted to use only during the implementation period of the TCS. Upon study completion, all physical and digital contact lists were destroyed and archived on record. Furthermore, participant information was strictly handled in an anonymized form and used solely for academic research purposes.

Participants received a gift card worth NT $100 (approximately US $3.2) upon completion of the survey as a token of appreciation. No additional compensation, insurance, or risk-related arrangements were deemed necessary due to the minimal risk nature of school-based data collection.

### Research Design

The predicted number of smartphone users in Taiwan is expected to reach 20.81 million by 2029, representing an increase of 0.7 million individuals [71].

The TCS project has been using structured questionnaire surveys and interview methods at the national level since 2012. The data used in this study were extracted from the TCS database available through the website [72] to learn the trend of media usage, social engagement, and social capital concern. TCS is a research program supported by the Government’s Ministry of Science and Technology to conduct an annual survey of the population with representative samples. It centers on monitoring shifts in consumption media patterns. Besides media use data analysis, the survey uses several specialized modules to discover current trends in health communication.

### Procedure

The TCS cohort project used a mixed methods approach, combining quantitative and qualitative interviews to comprehensively examine young users’ online information-seeking behaviors. The TCS report indicates that the sample selection included a multistage and stratified random methodology to guarantee a representative cross-section of participants [73]. The approach involved a substantial number of students who completed a survey, offering insights into their preferences, sources, and motivations for accessing online knowledge. In a subsequent study, certain respondents were invited to participate in qualitative interviews, facilitating a more profound examination of their experiences and perceptions regarding online information.

The TCS project and data collection received approval from the members of the Taiwan Institutional Review Board before implementation. All data were gathered from September to October 2020. Trained supervisors who conducted the survey were mandated to visit the sampling classes at 62 schools across the nation.

A total of 1696 respondents aged 9-18 years included 19 elementary schools with 446 valid surveys and 20 junior high schools with 582 valid responses. A total of 15 secondary schools and 8 technical high schools were selected for sampling from high school students. A total of 668 surveys were completed. Data from participants aged 9-18 years in the 2020 TCS project were collected. To maintain analytical consistency aligned with the study’s objective, individuals aged 10-18 years were examined in this research (n=1673). The research achieved a confidence level of 95%, with a sampling error not exceeding 2.38%.

### Measurements

#### Parental Monitoring

This analysis examines the potential influences on the relationship between parental monitoring and mobile phone addiction, focusing on PSU, parental monitoring, and demographic factors. Parental mediation consists of 3 components: restrictive mediation (questions 1 to 3, Cronbach α coefficient=0.761), evaluative mediation (questions 5 and 6, Cronbach α coefficient=0.674), and unfocused mediation (question 4). Restrictive mediation encompasses parental limitations on specific websites, regulations regarding media consumption, and imposed time constraints. Evaluative mediation entails parents engaging with media and subsequently discussing their usage and experiences with their children. Unfocused mediation denotes a parental approach in which children are encouraged to use media without any form of guidance or limitations.

#### PSU

The evaluation of mobile phone addiction was conducted using 5 questions that used a 5-point scale, ranging from “I strongly disagree” to “I strongly agree.” The final PSU score represents the average (mean) of the 5 items, where higher scores indicate greater levels of mobile phone addiction. These questions were designed to evaluate the participants’ behavior and their views on the use of mobile phones. Items assessed loss of control, functional impairment, and preoccupation (eg, “You daydream about your phone”). The formation of these 5 questions was done following previous studies on PSU [74]. The purpose of these questions was to assess the participants’ behaviors and views regarding mobile phones. The Cronbach α coefficient for 5 questions is 0.769, and the McDonald omega is 0.788.

#### Sociodemographic Characteristics

The demographic data included age, gender, school grade, parental educational achievement, family income level, and marital status. In addition to descriptive results, multiple linear regression analysis and moderated multiple serial mediation analyses unveiled the substantial predictive capacity of sociodemographic characteristics and general parental supervision in shaping internet usage and effects on mobile phone addiction. Concretely, the regression models explained how and to what extent each of the demographic variables influenced the relationship between parental monitoring and mobile phone addiction. Demographic variables like age, gender, parents’ education levels, and household income were identified as significant factors influencing individuals’ internet usage, PSU, and parent monitoring.

This comprehensive methodology, involving meticulous sampling, data collection over various time periods in 2020, and advanced statistical analysis, serves as the foundation for understanding the intricate relationship between demographic factors and online information-seeking behavior within the context of the specified information categories. The complete list of variables, their descriptions, question phrasing, and scaling details can be found in Multimedia Appendix 1.

#### Data Processing

The statistical software IBM SPSS 29 was used to perform both descriptive and regression analyses of the collected data. Descriptive statistics were used as measures of overview tendencies to give general information regarding the frequencies and general patterns regarding internet usage, mobile phone addiction, and parental supervision among adolescents.

Besides descriptive analysis, multiple linear regression analysis, and moderated multiple serial mediation analyses unveiled the substantial predictive capacity of sociodemographic characteristics and general parental supervision in shaping internet usage and effects on smartphone addiction. Concretely, the regression models explained how and to what extent each of the demographic variables—age, gender, parents’ education levels, and household income—influenced the relationship between parental monitoring and smartphone addiction.

## Results

### Descriptive Statistics

An analysis of internet and digital device usage patterns was performed among children and teenagers in 2020. A total of 1673 valid data were evaluated. It is found that 92.3% (n=1545) of children and teenagers accessed the internet in Taiwan, among the respondents, with a mean age of 13.31 (SD 2.294) years. Out of the total number of respondents, 51.8% (n=867) were male and 48.2% (n=806) were female. A significant proportion of the participants’ parents possessed a high school diploma or a higher level of education, specifically 29.2% (n=488) and 27% (n=451), respectively, with a total sample size of 939. Approximately 73.7% of respondents, namely 1233 individuals, believe that their household income level is relatively affluent. Among adolescents, 83.9% (n=1423) own mobile phones and 92% (n=1561) use their mobile phones to access the internet. The mean score of PSU was 3.29 (SD 0.84). The descriptive results for parental monitoring revealed that restrictive mediation had a mean score of 2.39 (SD 0.90), followed by evaluative mediation with a mean of 2.22 (SD 0.86), and unfocused mediation with a mean of 2.11 (SD 0.95). Multimedia Appendix 2 provides the statistical information for adolescent respondents from Taiwan in 2020.

### Effects of Parental Monitoring

Among the 3 types of parental monitoring, restrictive monitoring was the most effective. This approach had a significant indirect effect on mobile phone addiction through decreased internet usage (b=−0.029, SE 0.007, 95% CI −0.044 to −0.017). The direct effect of restrictive monitoring on phone addiction (controlling for internet use) was significant (b=−0.154, SE 0.024, *P*<.001). Further analysis showed that restrictive monitoring significantly decreased internet usage (b=−0.338, SE 0.036, *P*<.001). These findings confirm that reducing screen time is a key pathway by which strict parental rules help lower mobile phone dependency.

In contrast, evaluative monitoring, which involves discussing media use with teens, had mixed effects. It was associated with slightly higher internet use (b=0.090, SE 0.038, *P*=.02), but its direct effect on phone addiction was not statistically significant (b=−0.037, SE 0.024, *P*=.13). This suggests that while open communication may encourage greater digital engagement, it does not necessarily reduce problematic mobile phone use.

Unfocused monitoring, where parents offer little structure or guidance, was linked to higher internet use (b=0.145, SE 0.035, *P*<.001) but showed no significant effect on mobile phone addiction (b=−0.010, SE 0.022, *P*=.66). This indicates that inconsistent or vague monitoring is ineffective at managing digital behavior.

These findings partially support the first hypothesis that parental monitoring helps prevent mobile phone addiction among adolescents. Concretely, restrictive monitoring was the most effective in reducing both internet use and phone addiction. While restrictive monitoring was protective, both evaluative and unfocused monitoring were associated with increased internet use, which in turn was linked to higher levels of PSU. Table 1 provides the details of the regression analysis illustrating the impact of parental monitoring and internet usage on mobile phone addiction.

**Table 1.** The bootstrap results of the moderated multiple serial mediation model.

Path	Mobile phone addiction (Y), b (SE)	*t* (*df*)	*P* value	Bootstrapped 95% CI	
Parent monitor_ restrictive (X) n=1584					
Direct effect (X→Y)	−0.154 (0.024)	−6.477 (1582)	<.001	−0.201 to −0.108	
Direct effect (X→internet use)	−0.338 (0.036)	−9.340 (1582)	<.001	−0.408 to −0.267	
Indirect effect (X→internet use→Y)	−0.029 (0.007)	—^a^	—	−0.044 to −0.017	
Parent monitor_ evaluative (X) n=1584					
Direct effect (X→Y)	−0.037 (0.024)	−1.515 (1582)	.13	−0.084 to 0.011	
Direct effect (X→internet use)	0.090 (0.038)	2.365 (1582)	.02	0.015 to 0.165	
Indirect effect (X→internet use→Y)	0.010 (0.005)	—	—	0.001 to 0.020	
Parent monitor_ unfocused (X) n=1584					
Direct effect (X→Y)	−0.010 (0.022)	−0.445 (1582)	.66	−0.053 to 0.034	
Direct effect (X→internet use)	0.145 (0.035)	4.207 (1582)	<.001	0.078 to 0.213	
Indirect effect (X→internet use→Y)	0.016 (0.005)	—	—	0.008 to 0.026	

a Not applicable.

### Variable of Sex

Sex does not moderate the effect of any form of parental monitoring; for instance, restrictive monitoring, internet use, and mobile phone addiction (b=0.0008, SE 0.009, 95% CI −0.019 to 0.019). When sex was introduced into the analysis, it was revealed that it had no effect on the relationship between the level of parental monitoring, internet use, and mobile phone addiction. Thus, it was found that there was a lack of sex differences in terms of perceived parental monitoring, internet use, and mobile phone addiction. This result, illustrated in Table 2, failed to reject the null hypothesis of the second hypothesis, which proposed that girls were likely to experience more parental monitoring than boys in the use of the internet and mobile phone addiction.

**Table 2.** The bootstrap results of the moderated multiple serial mediation of sex and age for mobile phone addiction.

Path	Mobile phone addiction (Y), b (SE)	*t *(*df*)	*P* value	Bootstrapped 95% CI	
Parent monitor_ restrictive (X)^a^ n=1584					
Direct effect	−0.163 (0.036)	−4.522 (1582)	<.001	−0.2234 to −0.092	
Indirect effect (X→internet use→Y)	0.0008 (0.009)	—^b^	—	−0.019 to 0.019	
Parent monitor_ evaluative (X)^c^ n=1584					
Direct effect	−0.101 (0.038)	−2.633 (1582)	.008	−0.177 to −.026	
Indirect effect (X→internet use→Y)	0.019 (0.013)	—	—	−0.007 to 0.046	
Parent monitor_ unfocused (X)^d^ n=1584					
Direct effect	−0.053 (0.033)	−1.588 (1582)	.11	−0.120 to 0.012	
Indirect effect (X→internet use→Y)	0.019 (0.011)	—	—	−0.003 to 0.043	

a X* Gender (W): *R*2=0.001, *P*=.14, *F*=2.164.

b Not applicable.

c X* Gender (W): *R*2=0.0002, *P*=.60, *F*=0.282.

d X* Gender (W): R2=0.001, *P*=.18, *F*=1.830.

### Variable of Age

The influence of internet usage on PSU differed depending on the age group. Internet use was found to be a significant predictor of mobile phone addiction among teenagers aged 10-14 years. However, this prediction was not significant for those aged 15-18 years. Among 10-year-olds, heightened unfocused parental monitoring had a significant negative effect on mobile phone addiction (b=−0.333, SE 0.160, *t*_51_=−2.084, *P*=.04). This indicates that unfocused parental monitoring can lower mobile phone addiction, but it did not have a significant influence on internet usage.

Concomitantly, 11-year-old users’ heightened restrictive parental monitoring led to a substantial decrease in internet usage and mobile phone addiction (b=−0.066, SE 0.028, 95% CI −0.129 to −0.019). Furthermore, restrictive monitoring also has a negative direct impact on the development of mobile phone addiction (b=−0.185, SE 0.085, *t*_189_=−2.173, *P*=.03). Additionally, evaluative monitoring negatively correlated with mobile phone addiction (b=−0.164, SE 0.071, *t*_189_=−2.322, *P*=.02). In contrast, unfocused mediation did not exert a substantial direct impact on mobile phone addiction.

Among 12-year-olds, implementing stricter parental monitoring (restrictive monitoring) resulted in a substantial decrease in internet usage (b=−0.033, SE 0.017, 95% CI −0.073 to −0.038). However, restrictive parental monitoring did not have a significant effect on mobile phone addiction. On the other hand, when parents are using unfocused monitoring, there is a substantial rise in internet usage. Additionally, higher levels of internet usage are associated with a greater likelihood of developing mobile phone addiction (b=0.042, SE 0.018, 95% CI 0.011-0.083).

Among 13-year-olds, stricter parental monitoring (restrictive monitoring) was found to be directly linked to a decrease in mobile phone addiction (b=−0.240, SE 0.073, *t*_226_=−3.288, *P*=.001). Users among 16-year-olds with heightened unfocused parental monitoring have a significant negative impact on internet usage. However, unfocused did not have a significant effect on mobile phone addiction (b=−0.004, SE 0.007, 95% CI −0.022 to 0.008).

The findings support hypothesis 3, indicating that younger adolescents benefit more from restrictive parental monitoring in reducing both internet usage and mobile phone addiction, while older adolescents are less affected by such monitoring techniques. The results imply that there are differences in various types of parental supervision regarding the age of the child. The effects of parental oversight are not uniform on both internet use and mobile phone dependency, which answered the RQ2. Table 3 displays the overview of results for the significant result of synergy of the mediation effects for age groups 10 to 13 and 16 as identified in this study.

**Table 3.** Overview of the bootstrap results of the moderated multiple serial mediation for mobile phone addiction.

Path	Mobile phone addiction (Y), b (SE)	*t* (*df*)	*P* value	Bootstrapped 95% CI
10-year-old (n=53)				
Parent monitor_Restrictive (X)				
Direct effect	−0.174 (0.164)	−1.064 (51)	.29	−0.504 to 0.155
Indirect effect (X→internet use→Y)	−0.092 (0.072)	—^a^	—	−0.246 to 0.046
Parent monitor_Evaluative (X)				
Direct effect	−0.153 (0.168)	−0.908 (51)	.37	−0.492 to 0.185
Indirect effect (X→internet use→Y)	0.084 (0.104)	—	—	−0.029 to 0.364
Parent monitor_Unfocused (X)				
Direct effect	−0.333 (0.160)	−2.084 (51)	.04	−0.655 to −0.012
Indirect effect (X→internet use→Y)	0.077 (0.063)	—	—	−0.070 to 0.188
11-year-old (n=191)				
Parent monitor_Restrictive (X)				
Direct effect	−0.185 (0.085)	−2.173 (189)	.03	−0.354 to −0.017
Indirect effect (X→internet use→Y)	−0.066 (0.028)	—	—	−0.129 to −0.019
Parent monitor_Evaluative (X)				
Direct effect	−0.164 (0.071)	−2.322 (189)	.02	−0.304 to 0.024
Indirect effect (X→internet use→Y)	0.0004 (0.021)	—	—	−0.042 to 0.046
Parent monitor_Unfocused (X)				
Direct effect	0.008 (0.066)	0.122 (189)	.90	−0.122 to 0.139
Indirect effect (X→internet use→Y)	0.036 (0.023)	—	—	−0.0008 to 0.089
12-year-old (n=303)				
Parent monitor_Restrictive (X)				
Direct effect	−0.076 (0.073)	−1.045 (301)	.30	−0.221 to 0.067
Indirect effect (X→internet use→Y)	−0.033 (0.017)	—	—	−0.073 to −0.003
Parent monitor_Evaluative (X)				
Direct effect	−0.089 (0.063)	−1.397 (301)	.16	−0.214 to 0.036
Indirect effect (X→internet use→Y)	0.011 (0.012)	—	—	−0.010 to 0.038
Parent monitor_Unfocused (X)				
Direct effect	−0.133 (0.063)	−2.112 (301)	.04	−0.258 to 0.009
Indirect effect (X→internet use→Y)	0.042 (0.018)	—	—	0.011 to 0.083
13-year-old (n=228)				
Parent monitor_Restrictive (X)				
Direct effect	−0.240 (0.073)	−3.288 (226)	.001	−0.384 to −0.096
Indirect effect (X→internet use→Y)	−0.013 (0.014)	—	—	−0.047 to 0.010
Parent monitor_Evaluative (X)				
Direct effect	−0.102 (0.080)	−1.275 (226)	.20	−0.259 to 0.055
Indirect effect (X→internet use→Y)	0.019 (0.019)	—	—	−0.010 to 0.068
Parent monitor_Unfocused (X)				
Direct effect	−0.004 (0.069)	−0.060 (226)	.95	−0.141 to 0.132
Indirect effect (X→internet use→Y)	0.023 (0.014)	—	—	−0.0003 to 0.054
16-year-old (n=281)				
Parent monitor_Restrictive (X)				
Direct effect	−0.069 (0.069)	−0.993 (279)	.32	−0.206 to 0.068
Indirect effect (X→internet use→Y)	−0.003 (0.007)	—	—	−0.020 to 0.010
Parent monitor_Evaluative (X)				
Direct effect	−0.065 (0.058)	−1.129 (279)	.26	−0.180 to 0.048
Indirect effect (X→internet use→Y)	0.0003 (0.004)	—	—	−0.007 to 0.009
Parent monitor_Unfocused (X)				
Direct effect	0.026 (0.052)	0.500 (279)	.62	−0.076 to 0.128
Indirect effect (X→internet use→Y)	−0.004 (0.007)	—	—	−0.022 to 0.008

a Not applicable.

### Household Income and Parents’ Education

Parental use of restrictive monitoring is influenced by 2 main factors: household income and the mother’s education level. Higher-income families are more likely to adopt restrictive strategies, which effectively reduce adolescent smartphone addiction. The analyses revealed that the mediator (restrictive mediation) and income (X) were significant predictors of mobile phone addiction (b=−0.022, SE 0.010, 95% CI −0.043 to −0.003). It is found that households with higher incomes tend to use restrictive control measures, which successfully reduce the likelihood of cell phone addiction among adolescents.

The findings also show that higher-income families are more likely to use evaluative mediation, which involves discussing phone use with their children. This approach was positively associated with wealth (b=0.189, SE 0.037, *t*_1582_=5.060, *P*<.001). Similarly, there was a positive association between higher household income (b=0.092, SE 0.041, *t*_1582_=2.228, *P*=.03) and increased use of unfocused mediation. The findings indicate that specific socioeconomic characteristics have an impact on the evaluative and unfocused type of parental supervision used, but they do not have an indirect influence on the decreasing probability of adolescent mobile phone addiction. The third RQ delves into the intricate relationship between income, mobile phone addiction, and internet usage patterns. Table 4 displays the results of the third RQ and fourth hypothesis, which answered the mediation relationship between income, mobile phone addiction, and restrictive parental monitoring.

**Table 4.** The bootstrap results of income of the moderated multiple serial mediation model.

Path (income [X], n=1584)	Mobile phone addiction (Y), b (SE)	*t* (*df*)	*P* value	Bootstrapped 95% CI
PM_restrictive				
Direct effect (X →Y)	−0.087 (0.040)	−2.154 (1582)	.03	−0.166 to −0.078
Direct effect (X →PM_restrictive)	0.092 (0.038)	2.395 (1582)	.02	0.016 to 0.168
Indirect effect (X →PM_restrictive→Y)	−0.022 (0.10)	—^a^	—	−0.043 to −0.003
PM_evaluative				
Direct effect (X →Y)	−0.100 (0.041)	−2.394 (1582)	.02	−0.182 to −0.018
Direct effect (X →PM_restrictive)	0.189 (0.037)	5.060 (1582)	<.001	0.115 to 0.262
Indirect effect (X →PM_evaluative→Y)	−0.009 (0.006)	—	—	−0.022 to 0.001
PM_unfocused				
Direct effect (X →Y)	−0.110 (0.041)	−2.666 (1582)	.007	−0.192 to −0.029
Direct effect (X →PM_restrictive)	0.092 (0.041)	2.228 (1582)	.03	0.011 to 0.173
Indirect effect (X →PM_unfocused→Y)	0.001 (0.002)	—	—	−0.004 to 0.007

a Not applicable.

Mother’s education was found to influence adolescent smartphone addiction. Higher maternal education was linked to lower phone addiction (b=−0.001, SE 0.0007, *t*_1582_=−2.709, *P*=.006), and it also predicted greater use of restrictive parental monitoring (b=0.001, SE 0.0006, *t*_1582_=2.879, *P*=.004). Thus, it is reported that mothers who have greater levels of education tend to use restrictive meditation practices, which successfully reduce the likelihood of kids developing cell phone addiction. Also, children of mothers with greater levels of education are less likely to develop mobile phone addiction (b=−0.0004, SE 0.0002, 95% CI −0.008 to −0.0001). RQ4 was solved based on this finding, and the result is presented in Table 5.

**Table 5.** The bootstrap results of parental education of the moderated multiple serial mediation model.

Path	Mobile phone addiction (Y), b (SE)	*t* (*df*)	*P* value	Bootstrapped 95% CI
PM_restrictive				
Father education (X) n=1584				
Direct effect (X →Y)	−0.0004 (0.0006)	−.6404 (1582)	.52	−0.001 to 0.0008
Direct effect (X →PM_restrictive)	0.002 (0.0006)	4.384 (1582)	<.001	0.001 to 0.003
Indirect effect (X →PM_restrictive→Y)	−0.0006 (0.0002)	—^a^	—	−0.001 to 0.0003
Mother education (X) n=1584				
Direct effect (X →Y)	−0.001 (0.0007)	−2.709 (1582)	.006	−0.003 to 0.0005
Direct effect (X →PM_restrictive)	0.001 (0.0006)	2.879 (1582)	.004	0.0006 to 0.003
Indirect effect (X →PM_restrictive→Y)	−0.0004 (0.0002)	—	—	−0.008 to −0.0001
PM_evaluative				
Father education (X) n=1584				
Direct effect (X →Y)	−0.001 (0.0007)	−1.909 (1582)	.06	−0.002 to 0.0001
Direct effect (X →PM_ evaluative)	−0.003 (0.0006)	−5.063 (1582)	<.001	−0.004 to −0.001
Indirect effect (X →PM_evaluative→Y)	0.0002 (0.0001)	—	—	−0.0001 to 0.0004
Mother education (X) n=1584				
Direct effect (X →Y)	−0.002 (0.0007)	−3.343 (1582)	<.001	−0.003 to −0.001
Direct effect (X →PM_ evaluative)	−0.002 (0.0006)	−4.181 (1582)	<.001	−0.003 to −0.001
Indirect effect (X →PM_evaluative→Y)	0.0002 (0.0001)	—	—	0.0001 to 0.0004
PM_unfocused				
Father education (X) n=1584				
Direct effect (X →Y)	−0.001 (0.0007)	−1.597 (1582)	.11	−0.002 to .0002
Direct effect (X →PM_ unfocused)	−0.002 (0.0006)	−3.406 (1582)	<.001	−0.003 to −0.0009
Indirect effect (X →PM_ unfocused→Y)	0.0001 (0.0001)	—	—	−0.0001 to 0.0001
Mother education (X) n=1584				
Direct effect (X →Y)	−0.002 (0.0007)	−3.207 (1582)	.001	−0.003 to −0.0009
Direct effect (X →PM_ unfocused)	−0.001 (0.0007)	−2.624 (1582)	.008	−0.003 to −0.0004
Indirect effect (X →PM_ unfocused→Y)	0.0001 (0.0001)	—	—	−0.0001 to 0.0001

a Not applicable.

The data also showed that parents with lower education levels, both fathers (b=−0.003, SE 0.0006, *t*_1582_=−5.063, *P*<.001) and mothers (b=−0.002, SE 0.0006, *t*_1582_=4.181, *P*<.001) were more likely to use evaluative mediation. Nevertheless, this type of mediation also had a measurable impact on reducing smartphone addiction. Meanwhile, lower parental education levels were associated with the use of unfocused mediation, both for fathers (b=−0.002, SE 0.0006, *t*_1582_=−3.406, *P*<.001) and mothers (b=−0.001, SE 0.0007, *t*_1582_=−2.624, *P*=.008). However, this technique was found to have little or no predictive value for cell phone addiction.

Hence, the study partially supports hypothesis 5, which hypothesizes that household income and parental education are the 2 main factors that influence the style of monitoring by parents, with only restrictive monitoring as being effective in preventing addiction to mobile phone use. However, the sociodemographic variables, such as income and parent education level, are often linked to evaluative and unfocused monitoring but have little impact on the likelihood of mobile phone addiction.

## Discussion

### Principal Results

This study examined the complex interplay between parental monitoring, adolescent development, and PSU. Findings demonstrated a strong association between parental supervision strategies and digital behaviors, corroborating previous research that links monitoring to reduced internet addiction [75,76]. Notably, restrictive mediation was more effective among younger adolescents, significantly curbing internet and smartphone use for those aged 10‐12 years. This supports earlier findings that younger children are more responsive to parental authority [53,55]. However, as adolescents matured, restrictive strategies proved less effective. According to past studies, older adolescents, particularly those aged 15‐18 years, demonstrated greater resistance to control [48,49,77]. With advanced problem-solving skills and critical thinking, they are able to manage their digital lives independently and evade parental detection [78].

Evaluative mediation, characterized by open dialogue and critical reflection, was associated with increased internet use; however, it did not significantly reduce problematic mobile phone use. Unfocused monitoring yielded inconsistent outcomes: among 11‐12-year-olds, it increased internet use, but by age 16, it was linked to reduced usage. These results underscore the developmental dimension of digital behavior regulation and emphasize the need for age-sensitive parental strategies.

Age emerged as a key moderator in the relationship between parental oversight and adolescent smartphone behavior. As cognitive maturity develops, adolescents increasingly prioritize peer affiliation and autonomy over parental control [79,80].

Consequently, the study reinforces that while restrictive strategies may work for younger cohorts, autonomy-supportive and collaborative forms of oversight are more effective for older adolescents. These findings directly inform SRSE-based interventions by identifying parental style as a modifiable determinant that mediates their effectiveness.

### Developmentally Adaptive Parental Mediation

The developmental trajectory of adolescence demands flexible parental mediation strategies [81]. Research findings suggest that parental oversight functions not merely as a passive background variable, but as a dynamic moderator that either enhances or inhibits SRSE-based interventions. When adolescents perceive parental control as autonomy-supportive, emphasizing trust, communication, and shared decision-making, they are more likely to internalize self-regulatory behaviors. In contrast, controlling or punitive oversight may provoke psychological reactance and lead to resistance, especially in older adolescents [48,49,77].

This aligns with parental mediation theory, which positions parenting as an adaptive, interactive process shaped by both the parent’s approach and the adolescent’s developmental stage. Integrating this with the PYD framework allows for a more holistic understanding of adolescent digital well-being. Rather than viewing smartphone use as inherently problematic, this perspective emphasizes fostering competencies, such as critical thinking and emotional regulation, that enable responsible digital engagement. Parental mediation strategies that evolve in tandem with adolescent cognitive and social maturity can effectively bridge these models, guiding healthier digital development.

### Socioeconomic Influences and Theoretical Implications

While the developmental stage significantly shaped the effectiveness of parental strategies, our findings also illuminated how socioeconomic variables influenced smartphone addiction and mediation style. Consistent with previous research, adolescents from low-income and lower-education households demonstrated higher levels of smartphone addiction [63-65]. These families often lack alternative recreational resources or digital literacy [66], which may lead to greater reliance on smartphones as surrogate caregivers. In contrast, high-income families, benefiting from greater resources and parental availability, implemented more diverse and effective mediation approaches, thereby reducing smartphone dependency.

Interestingly, while parental education was inversely related to adolescent smartphone addiction, it also influenced the choice of monitoring strategy. Parents with higher education tended to favor restrictive mediation, while parents with lower education leaned toward evaluative or unfocused approaches [69]. These findings indicate that effective digital parenting is shaped not only by knowledge and resources but also by attitudes and awareness regarding digital risks. Importantly, this supports the argument that interventions must account for both perceptual and structural determinants of parenting behavior.

Contrary to some earlier assumptions, this study found no significant sex differences in parental monitoring or smartphone use. Both male and female adolescents were subject to similar levels of parental oversight, aligning with research that documents diminishing sex-based parenting distinctions in the digital age [52,82]. This neutrality in parental response further emphasizes the relevance of focusing on developmental and contextual factors rather than demographic stereotypes when designing digital well-being interventions.

These results advance theoretical integration by situating parental mediation within a bidirectional model of digital development. The convergence of parental mediation theory with SRSE and PYD frameworks underscores the importance of tailoring interventions to the evolving cognitive, emotional, and environmental realities of adolescents. Parental oversight should be understood not as a fixed constraint but as an evolving, context-dependent factor with the potential to amplify or hinder the success of digital health initiatives.

This study used a nationally representative dataset from Taiwan, where adolescents aged 10‐18 years encounter one of the highest rates of digital saturation in East Asia. Its Confucian-influenced parenting norms and academic pressures create a culturally unique yet regionally relevant context.

Insights obtained from the Taiwanese context can therefore enhance the comprehension of parental mediation in other industrialized East Asian nations, namely South Korea, Japan, and Singapore, which possess analogous sociocultural and educational attributes. In both settings, the efficacy of parental mediation measures is significantly affected by developmental stage and perceived autonomy, rendering Taiwan a crucial case study for comparative digital parenting research.

### Limitations

Despite offering valuable insights into adolescent digital behavior and parental supervision, this study has several limitations. Primarily, the data were self-reported, which introduces potential biases such as social desirability and recall errors [83,84]. Previous research involving 3401 participants shows that only one-third of respondents accurately report internet usage, while 42% (n=1428) overestimate and 26% (n=884) underestimate it [85]. Heavy users tend to underreport, whereas light users often overreport [86]. Such inaccuracies can distort key behavioral metrics, especially when relying on survey-based primary data.

Furthermore, the COVID-19 pandemic likely influenced both adolescent behavior and parental monitoring. Shifting family routines, including remote work and increased household responsibilities, may have altered parents’ capacity to supervise screen time effectively [87]. As families transitioned back to prepandemic structures, supervision patterns likely changed, complicating interpretations of consistency over time.

This study did not directly measure smartphone ownership, which recent research suggests can be a significant moderator of parental mediation efficacy and associated child outcomes [88]. In our high-penetration context (94.9%), ownership variance was minimal, likely limiting its moderating effect within our sample. However, this limits the generalizability of our findings to cultures or socioeconomic contexts where smartphone access among adolescents is not near-universal. Future studies should include this variable to better elucidate its role across different environments.

Additionally, the measurement of parental mediation was constrained by the limited number of items in each subdimension, especially unfocused mediation. This restricts the internal consistency of the overall scale and limits the interpretive power of the unfocused mediation category, which cannot yield a reliability estimate.

Future research should incorporate both subjective and objective digital usage metrics, include perspectives from both adolescents and parents, and consider cultural and demographic contexts. Longitudinal approaches would better capture evolving behavioral dynamics and improve the reliability of assessments across diverse environments.

### Conclusions

This study investigated how parental monitoring shapes adolescents’ digital well-being in Taiwan, using data from the nationwide survey. The findings highlight the critical role of age-specific parental strategies in mitigating excessive internet use and mobile phone addiction. Restrictive monitoring proved effective for younger adolescents, but its influence declined with age, emphasizing the need for adaptive, communication-centered approaches as adolescents develop greater autonomy.

As digital devices become integral to education and social interaction, especially in the postpandemic context, balancing screen use with psychosocial health is increasingly complex. Our results suggest that instead of relying solely on rigid restrictions, parents should promote constructive digital engagement, particularly among older adolescents. Equipping adolescents with self-regulatory skills through open dialogue and guidance may prove more effective in fostering responsible smartphone use.

While this research focuses on Taiwanese adolescents, its findings may resonate across culturally similar East Asian contexts. Taiwan’s high smartphone penetration, academic intensity, and Confucian-rooted family dynamics make it a meaningful case study for digital parenting in developed Asian societies. Thus, the insights gained from this study are not only locally relevant but regionally informative, contributing to broader comparative understandings of adolescent digital regulation in Asia.

This research carries implications for families, educators, and policymakers. Awareness campaigns and parental support programs—particularly targeting lower-income and less-educated households—are essential in promoting effective mediation strategies. Community institutions, such as schools, can serve as platforms for delivering digital literacy interventions that empower families to navigate adolescents’ digital lives with confidence and care. Future efforts should aim to develop developmentally sensitive, culturally attuned tools that support both youth well-being and healthy digital integration.

## Supplementary material

10.2196/75837 Multimedia Appendix 1 Detailed information about measurements.

10.2196/75837 Multimedia Appendix 2 Descriptive statistics of Taiwanese adolescent respondents in 2020.

## Abbreviations

PSU: problematic smartphone use
PYD: positive youth development
RQ: research question
SRSE: self-regulated smartphone engagement
TCS: Taiwan Communication Survey
