# Supplementary material for: Exploring the Impact of Perceived Parental Oversight on Problematic Smartphone Use Among Adolescents in the Digital Age: Database Analysis
Source: JMIR Pediatr Parent. 2025 Dec 4;8:e75837. doi: 10.2196/75837 (PMC12677870; doi:10.2196/75837)
Supplement: Multimedia Appendix 1 [file pediatrics-v8-e75837-s001.docx]

## Multimedia Appendix 1

Detailed information about measurements.

| **Mobile phone addiction** (1 = strongly disagree; 5 = strongly agree) | | | |
| --- | --- | --- | --- |
| - You find yourself using your phone absent-mindedly. | | | |
| - You realized you checked your phone only after you have already been using it. | | | |
| - You daydream about your phone. | | | |
| - You neglect work or schoolwork to spend more time using your phone. | | | |
| - You should decrease the amount of time using your phone. | | | |
| **Parental monitor** (1 = Never; 4 = Often) | | | |
| In this section, we would like to know how you use the Internet at home. Please respond based on actual conditions. | | | |
| - My parents don’t allow me to visit certain websites. | | | |
| - My parents set rules about when I can use the Internet and when I cannot. | | | |
| - My parents set a limit on how long I use the Internet. | | | |
| - My parents encourage me to use the Internet. | | | |
| - My parents use the Internet and discuss Internet use and experiences with me. | | | |
| - My parents discuss online stories and events with me. | | | |
| **Use of Internet** (Fill in blank.) | | | |
| - How many days per week on average do you surf the Internet using a computer (excluding the usage of mobile phones and tablets; only your Internet-surfing screen time)? | | | |
| - How many days per week on average do you surf the Internet using a tablet (only your Internet-surfing screen time)? | | | |
| - How many days per week on average do you surf the Internet using a mobile phone (e.g., Using LINE, watching YouTube) (only your Internet-surfing screen time)? | | | |
| **Use of mobile phone** (Fill in blank.) | | | |
| - On the days (only weekdays) that you’re using your cell phone, roughly how much time do you use it per day if you only count the time when you’re actually using the phone? _________ hour(s)_________ minute(s) | | | |
| - On the days that you’re using your cell phone on the weekend, roughly how much time do you use it per day if you only count the time when you’re actually using the phone? _________ hour(s)_________ minute(s) | | | |
| - How long do you surf the Internet using the following devices per day on average? (Ask about work and leisure separately.) How long do you surf the Internet using a mobile phone (only including your-Internet surfing screen time) for learning and working purposes every day? _____hours______minutes | | | |
| - How long do you surf the Internet using the following devices per day on average? (Ask about work and leisure separately.) Excluding your working and learning time online, how long do you surf the Internet using a mobile phone for entertainment and leisure purposes every day on average?_____hours_____minutes | | | |
| **Sociodemographic Variables** | | | |
| ***Gender*** | | | |
| - Male | | - Female | |
| ***Age*** (Fill in blank.) | | | |
| ***Father’s/ Mother’s Education*** | | | |
| - literate but no formal education | | - elementary school | |
| - junior high school | | - senior high school(vocational class) | |
| - junior college(two-year junior college, three-year junior college, five-year junior college) | | | |
| - university | | | |
| - graduate school(master’s degree, doctorate degree) | | | |
| - Other (please specify)______ | | | |
| - I don’t know | | | |
| ***Income*** | | | |
| How would you judge your family’s economic conditions? | | | |
| - Extremely poor | - Poor | | - Average |
| - Wealthy | - Extremely Wealthy | |  |
