# Supplementary material for: Exploring the Impact of Perceived Parental Oversight on Problematic Smartphone Use Among Adolescents in the Digital Age: Database Analysis
Source: JMIR Pediatr Parent. 2025 Dec 4;8:e75837. doi: 10.2196/75837 (PMC12677870; doi:10.2196/75837)
Supplement: Multimedia Appendix 2 [file pediatrics-v8-e75837-s002.docx]

## Multimedia Appendix 2

**Table 6.** Descriptive statistics of Taiwanese adolescent respondents in 2020.

| Variables (N = 1673) | | Frequency (%) |
| --- | --- | --- |
| Gender (n, %) | | |
|  | Male | 867 (51.8%) |
|  | Female | 806 (48.2%) |
| Age (M = 13.31, SD = 2.294) | | |
|  | 10-year-old | 130 (7.7%) |
|  | 11-year-old | 371 (22.2%) |
|  | 12-year-old | 273 (16.3%) |
|  | 13-year-old | 196 (11.7%) |
|  | 14-year-old | 97 (5.8%) |
|  | 15-year-old | 191 (11.7%) |
|  | 16-year-old | 251 (15.0%) |
|  | 17-year-old | 130 (7.8%) |
|  | 18-year-old | 33 (2.0%) |
| Mother’s Education | | |
|  | Primary school and below | 38 (2.3%) |
|  | Junior middle school | 104 (6.2%) |
|  | High school | 488 (29.2%) |
|  | Junior college | 214 (12.8%) |
|  | Bachelor’s degree | 365 (21.8%) |
|  | Higher Bachelor’s degree | 131 (7.8%) |
|  | Don’t know | 334 (19.9%) |
| Father’s Education | | |
|  | Primary school and below | 25 (1.4%) |
|  | Junior middle school | 167 (10.0%) |
|  | High school | 451 (27.0%) |
|  | Junior college | 157 (9.4%) |
|  | Bachelor’s degree | 316 (18.9%) |
|  | Higher Bachelor’s degree | 189 (11.3%) |
|  | Don’t know | 367 (22.0%) |
| Family’s economic conditions | | |
|  | Very poor | 15 (0.9%) |
|  | Poor | 136 (8.1%) |
|  | Relatively well-off | 1233 (73.7%) |
|  | Rich | 262 (15.7%) |
|  | Very rich | 27 (1.6%) |
